# Using imputation-based whole-genome sequencing data to improve the accuracy of genomic prediction for combined populations in pigs

**DOI:** 10.1186/s12711-019-0500-8

**Published:** 2019-10-21

**Authors:** Hailiang Song, Shaopan Ye, Yifan Jiang, Zhe Zhang, Qin Zhang, Xiangdong Ding

**Affiliations:** 1 Key Laboratory of Animal Genetics and Breeding of Ministry of Agriculture, National Engineering Laboratory of Animal Breeding, College of Animal Science and Technology, China Agricultural University, Beijing, China; 2 Guangdong Provincial Key Lab of Agro-Animal Genomics and Molecular Breeding, National Engineering Research Centre for Breeding Swine Industry, College of Animal Science, South China Agricultural University, Guangzhou, Guangdong China; 3 Shandong Provincial Key Laboratory of Animal Biotechnology and Disease Control and Prevention, Shandong Agricultural University, Taian, China

## Abstract

**Background:**

For genomic selection in populations with a small reference population, combining populations of the same breed or populations of related breeds is an effective way to increase the size of the reference population. However, genomic predictions based on single nucleotide polymorphism (SNP)-chip genotype data using combined populations with different genetic backgrounds or from different breeds have not shown a clear advantage over using within-population or within-breed predictions. The increasing availability of whole-genome sequencing (WGS) data provides new opportunities for combined population genomic prediction. Our objective was to investigate the accuracy of genomic prediction using imputation-based WGS data from combined populations in pigs. Using 80K SNP panel genotypes, WGS genotypes, or genotypes on WGS variants that were pruned based on linkage disequilibrium (LD), three methods [genomic best linear unbiased prediction (GBLUP), single-step (ss)GBLUP, and genomic feature (GF)BLUP] were implemented with different prior information to identify the best method to improve the accuracy of genomic prediction for combined populations in pigs.

**Results:**

In total, 2089 and 2043 individuals with production and reproduction phenotypes, respectively, from three Yorkshire populations with different genetic backgrounds were genotyped with the PorcineSNP80 panel. Imputation accuracy from 80K to WGS variants reached 92%. The results showed that use of the WGS data compared to the 80K SNP panel did not increase the accuracy of genomic prediction in a single population, but using WGS data with LD pruning and GFBLUP with prior information did yield higher accuracy than the 80K SNP panel. For the 80K SNP panel genotypes, using the combined population resulted in a slight improvement, no change, or even a slight decrease in accuracy in comparison with the single population for GBLUP and ssGBLUP, while accuracy increased by 1 to 2.4% when using WGS data. Notably, the GFBLUP method did not perform well for both the combined population and the single populations.

**Conclusions:**

The use of WGS data was beneficial for combined population genomic prediction. Simply increasing the number of SNPs to the WGS level did not increase accuracy for a single population, while using pruned WGS data based on LD and GFBLUP with prior information could yield higher accuracy than the 80K SNP panel.

## Background

Genomic prediction (GP), a method proposed by Meuwissen et al. [1], in which breeding values are predicted using dense genome-wide markers, has become a routine procedure in livestock breeding programs. In GP, quantitative trait loci (QTL) are presumed to be in linkage disequilibrium (LD) with at least one of the genotyped markers and the markers are used to estimate the level of genetic similarity between individuals and explain the genetic variance for the trait. Compared with pedigree-based prediction of breeding values, GP can be performed as soon as DNA is available, which allows accurate selection early in life. Generally, the accuracy of GP increases as the number of animals in the reference population increases. For small reference populations, combining populations of the same breed or populations of related breeds has been reported to increase the accuracy of GP, such as for Holstein populations in the EuroGenomics [2] and North American consortiums [3]. However, GP using combined populations has not shown a clear advantage compared with GP using a single population [4–6]. The reason may be that the populations that are combined are too divergent, such that LD between the QTL and genotyped SNPs is not sufficiently consistent between populations. Therefore, an important question in this regard is whether the accuracy of combined population GP can be improved by using whole-genome sequencing (WGS) markers instead of the lower-density SNP panels.

The availability of next-generation sequencing technologies has made it possible to apply GP to WGS data. Generally, increasing the number of SNPs in a panel increases the level of LD between a SNP and a QTL, which should be beneficial for GP. WGS data include a large number of genomic variants, including most causal mutations. Thus, prediction depends much less on LD between SNPs and causal mutations. However, some studies have demonstrated that using WGS data did not increase prediction accuracy or increased it only slightly compared to using high-density SNP panel genotypes. For example, van Binsbergen et al. [7] reported that using imputed WGS data did not increase the accuracy of GP in Holstein-Friesian cattle compared to using BovineHD SNP genotype data. Zhang et al. [8] also showed that increasing marker density did not increase or only slightly the accuracy of GP of feed efficiency component traits in Duroc pigs. Nevertheless, Brondum et al. [9] showed that the accuracy of GP could be improved by adding several significant QTL that were detected by genome-wide association studies (GWAS) using WGS data. Ni et al. [10] reported that using only SNPs in or around a gene from WGS data increased the accuracy of GP in laying chickens. Thus, GP with WGS data could be an attractive approach, although to date, the expectation of a higher accuracy has not been realized with real WGS data.

In pigs, most previous studies on GP have concentrated on populations with the same or very similar genetic backgrounds [11, 12]. However, in some national pig genetic improvement programs, pigs from a wide variety of sources are available, e.g., many Chinese pig breeding farms have no genetic links between them. To extend the size of the reference population, joint genetic evaluation can be performed by combining populations with different genetics, as in beef cattle [13, 14]. A previous study showed that using SNP genotypes from a combined reference population can improve the accuracy of GP, but this improvement was very small [6]. Using WGS data for combined population GP could be an attractive and meaningful approach to increase accuracy [15–17].

Our objective was to evaluate alternate approaches for combined population GP by analyzing WGS data. Three Yorkshire pig populations with different genetic backgrounds were used to establish a combined reference population, and two reproduction traits and two production traits were investigated to assess the accuracy of GP using different methods.

## Methods

### Ethics statement

The procedure for collecting pig blood samples was carried out in strict accordance with the protocol approved by the Animal Care and Use Committee of China Agricultural University (Permit Number: DK996).

### Populations and phenotypes

Yorkshire populations were sampled from three breeding farms in China, designated LM, XD, and ZX. Information on the populations and phenotypes is in Table 1. The pigs in the LM and ZX populations are both American Yorkshire progeny but from different breeding companies, and pigs in the XD population are British Yorkshire. The phenotypic records of the reproduction traits of piglets born alive (NBA) and total number of piglets born (TNB), and the production traits of days to 100 kg (AGE) and backfat thickness (BFT) were examined. BFT was measured between the 10th and 11th rib of pigs at a weight of ~ 100 kg by real-time B-mode ultrasound. AGE and BFT were measured from 85 to 130 kg, and then adjusted to 100 kg. AGE and BFT were calculated as follows: ${\text{AGE}} = {\text{measured}} \ {\text{age}} + \left( {100 - {\text{measured weight}}} \right) \times \frac{{{\text{measured age}}} - {{\text{CF}}}}{\text{measured weight}}$, where ${\text{CF}}$ is a correction factor (referring to the China National Swine Genetic Improvement Program) that is equal to 50.775 and 46.415 for males and females, respectively; ${\text{BFT}} = {\text{measured backfat thickness}} + \left( {100 - {\text{measured weight}}} \right) \times \frac{{\text{measured backfat thickness}}}{{{\text{measured weight}}} - {{\text{CF}}}}$, where ${\text{CF}}$ is equal to − 7.277 and − 9.440 for males and females, respectively. For the LM population, records were available for all reproduction and production traits, for the XD population only NBA and TNB records were available, and for the ZX population only production records were available.

**Table 1:** Summary of the three Yorkshire populations, numbers of genotyped animals, and estimates of heritability (${\text{h}}^{2}$)

Population^a^ (number of animals in the pedigree)	Origin	Trait	N-obs	Birth year	Genotyped animals	${\text{h}}^{2}$ (SE)
LM (72,998)	USA	NBA	5907/19,660	2004–2016	1641	0.08 (0.01)
		TNB	5907/19,660		1641	0.09 (0.01)
		AGE	28,827	2007–2016	1769	0.38 (0.02)
		BFT	28,827		1769	0.36 (0.02)
XD (51,964)	UK	NBA	4842/18,369	2004–2015	762	0.07 (0.01)
		TNB	4842/18,369		762	0.07 (0.01)
ZX (16,914)	USA	AGE	6721	2012–2016	320	0.19 (0.03)
		BFT	6721		320	0.24 (0.03)

*NBA* number of piglets born alive, *TNB* total number of piglets born, *AGE* days to 100 kg, *BFT* backfat thickness, *N-obs* number of individuals/observations

^a^Yorkshire populations from three Chinese pig breeding farms

### Derivation of corrected phenotypes

To avoid the double counting problem for parental information, corrected phenotypes that were derived from pedigree-based estimated breeding values (EBV) were used as response variables in GP analyses [8, 12]. The model used to estimate EBV is that defined by the Center of National Swine Genetic Evaluation of China (http://www.cnsge.org.cn/). Pedigree-based EBV and genetic parameters for reproduction traits were estimated based on a single-trait repeatability model, which was implemented separately for each population. In the model, the fixed effect included herd-year-season, in which season comprised four levels (1st = December to February; 2nd = March to May; 3rd = June to August; 4th = September to November), and the random effects included additive genetic, residual, and permanent environment effects. For the production traits, a bivariate animal model was implemented with the fixed effect of herd-year-season-sex and the random effects of additive genetics, litter, and residual. In total, 141,876 animals were traced back to construct a pedigree relationship matrix. Corrected phenotypic values ($y_{c}$) for reproduction were calculated as EBV plus the average of estimated residuals over parities for a sow, and as the EBV plus estimated residual for each individual for the production traits. Individuals with an EBV reliability lower than 0.3 were removed. The number of genotyped animals used in the study is in Table 1. EBV and EBV reliability were computed using the DMUAI procedure in the DMU software [18].

### Genotype data and imputation

Whole-genome sequence data on 289 pigs from 6 breeds were used as reference data for imputation, comprising 32 Duroc, 86 Large White, 29 Erhualian, 94 Yorkshire, 24 China South, and 24 China North pigs. All pigs had an average sequencing depth of ~ 25X and were introduced by Yan et al. [19] as a reference panel. SNP calling for these individuals was performed following the general next-generation sequencing data processing procedures, as described by Yan et al. [19]. After SNP calling, 46,766,110 SNPs were retained for imputation.

For the animals from the three populations used in this study, genomic DNA was extracted from blood samples using a TIANamp Blood DNA Kit (catalog number DP348; Tiangen, Beijing). Genotyping was performed using the PorcineSNP80 BeadChip (Illumina, San Diego, CA), which includes 68,528 SNPs across the pig genome. In total, 6103 pigs, which represented all families as best as possible, were genotyped as the target panel for imputation. Furthermore, 22 individuals in the target panel of 6103 pigs were sequenced (~ 10X) to calculate the genotype concordance rate (CR), which was defined as the proportion of genotypes of the imputed variants, which were the same as the whole-genome sequence variants.

Imputation from the 80K chip to WGS genotypes was performed with Beagle 4.1 [20]. Imputation accuracy was assessed by the allelic R-squared measure (AR2) in Beagle, which is an estimate of the squared correlation between the most probable and the true reference dose. Variants with a minor allele frequency (MAF) lower than 0.01 were excluded using the PLINK software (v1.90) [21] and only variants located on autosomes were used for further analysis, resulting in 56,463 SNPs from the 80K panel, and 18,976,288 SNPs for the imputed whole-genome sequence, henceforth referred to as WGS data. The following additional quality control criteria were used to remove SNPs from the WGS data: SNPs with an overall amplification of less than 90% and a random pair of SNPs that were in high LD with each other (r^2^ ≥ 0.9 [22]). After LD pruning, 4,099,253 SNPs were retained for the WGS data.

### Statistical models

Based on the 80K SNP data, the WGS data, and the pruned WGS data, three methods were used to predict breeding values using the corrected phenotypes: the GBLUP method based on the genomic relationship matrix, the single-step GBLUP (ssGBLUP) method with a combined pedigree–genomic relationship matrix, and the genomic feature BLUP (GFBLUP) method, which included an additional genomic effect that quantifies the joint effect of a group of variants associated with a genomic feature.

#### GBLUP

The GBLUP [23] model was used to predict the genomic EBV (GEBV) of all genotyped individuals:

$${\mathbf{y}}_{{\mathbf{c}}} = {\mathbf{1}}\upmu + {\mathbf{Zg}} + {\mathbf{e}},$$

where ${\mathbf{y}}_{{\mathbf{c}}}$ is the vector of corrected phenotypes, $\upmu$ is the overall mean, ${\mathbf{1}}$ is a vector of ones, ${\mathbf{g}}$ is the vector of genomic breeding values, following a normal distribution of ${\text{N}}({\mathbf{0}}, {\mathbf{G}}\sigma_{g}^{2}$), where $\sigma_{g}^{2}$ is the additive genetic variance, and ${\mathbf{G}}$ is the marker-based genomic relationship matrix [23]. ${\mathbf{Z}}$ is an incidence matrix linking ${\mathbf{g}}$ to ${\mathbf{y}}_{{\mathbf{c}}}$, and ${\mathbf{e}}$ is the vector of random errors, following a normal distribution of ${\text{N}}({\mathbf{0}}, {\mathbf{I}}\sigma_{e}^{2}$), where $\sigma_{e}^{2}$ is the residual variance.

#### ssGBLUP

The ssGBLUP model uses the phenotype information of both genotyped and non-genotyped animals in the same model as GBLUP, except that ${\mathbf{y}}_{{\mathbf{c}}}$ also contains the corrected phenotype values of the non-genotyped animals, and vector ${\mathbf{g}}$ is assumed to follow a normal distribution ${\text{N}}\left( {{\mathbf{0}}, {\mathbf{H}}\sigma_{g}^{2} } \right)$. The inverse of ${\mathbf{H}}$ is used in the mixed model equations, and is given by this simple form [24–26]:

$${\mathbf{H}}^{ - 1} = \left[ {\begin{array}{*{20}c} {{\mathbf{G}}_{{\mathbf{w}}}^{ - 1} - {\mathbf{A}}_{22}^{ - 1} } & {\mathbf{0}} \\ {\mathbf{0}} & {\mathbf{0}} \\ \end{array} } \right] + {\mathbf{A}}^{ - 1} ,$$

where ${\mathbf{A}}_{22}$ represents the submatrix of the pedigree-based relationship matrix ${\mathbf{A}}$ corresponding to the genotyped animals and, to avoid singularity problems, ${\mathbf{G}}_{{\mathbf{w}}} = 0.95{\mathbf{G}}_{\text{a}} + 0.05{\mathbf{A}}_{22}$ [27, 28], where ${\mathbf{G}}_{\text{a}}$ is an adjusted ${\mathbf{G}}$ according to Christensen et al. [11] to avoid differences in scale and location between the elements of ${\mathbf{G}}$ and elements of ${\mathbf{A}}_{22}$:

$${\mathbf{G}}_{a} = {\mathbf{G}}\upbeta +\upalpha\text{.}$$

where $\upalpha$ and $\upbeta$ are adjustment factors derived from the following equations:

$${\text{Avg}}. \, {\text{diag}}\left( {\mathbf{G}} \right)\upbeta +\upalpha = {\text{Avg}}.\, {\text{diag}}\left( {{\mathbf{A}}_{22} } \right)$$

$${\text{and}} \quad {\text{Avg}}.\, {\text{offdiag}}\left( {\mathbf{G}} \right)\upbeta +\upalpha = {\text{Avg}}.\, {\text{offdiag}}\left( {{\mathbf{A}}_{22} } \right),$$

where ${\text{Avg}}.\, {\text{diag}}$ is the average of the diagonal elements, and ${\text{Avg}}.\, {\text{offdiag}}$ is the average of the off-diagonal elements.

#### GFBLUP

The GFBLUP [29] model, which uses prior information about genomic features, is based on a linear mixed model with two random genomic effects:

$${\mathbf{y}}_{{\mathbf{c}}} = {\mathbf{1}}\upmu + {\mathbf{Zf}} + {\mathbf{Zr}} + {\mathbf{e}},$$

where ${\mathbf{y}}_{{\mathbf{c}}}$, ${\mathbf{1}}$, $\upmu$ and ${\mathbf{e}}$ are the same as in GBLUP, ${\mathbf{f}}$ is the vector of genomic values captured by genetic markers associated with a genomic feature of interest, following a normal distribution of ${\text{N}}({\mathbf{0}}, {\mathbf{G}}_{{\mathbf{f}}} \sigma_{f}^{2}$), ${\mathbf{r}}$ is the vector of genomic effects captured by the remaining set of genetic markers, following a normal distribution ${\text{N}}({\mathbf{0}}, {\mathbf{G}}_{{\mathbf{r}}} \sigma_{r}^{2}$), and ${\mathbf{Z}}$ is an incidence matrix that links ${\mathbf{f}}$ and ${\mathbf{r}}$ to ${\mathbf{y}}_{{\mathbf{c}}}$. Matrices ${\mathbf{G}}_{{\mathbf{f}}}$ and ${\mathbf{G}}_{{\mathbf{r}}}$ were constructed in the same way as ${\mathbf{G}}$, but using only the genetic marker set defined by a genomic feature, as described in the following, and the remaining markers, respectively. Variance components were estimated via the R package EMMREML (v3.1) [30].

Two strategies were used to define genetic markers that formed the different classes of genomic features used in GFBLUP model analyses. First, the following general linear model-based association analysis (GLMA) was conducted, as implemented in the MVP software [31]. After LD pruning, all WGS variants were tested for association:

$${\mathbf{y}}_{{\mathbf{c}}} = {\mathbf{1}}\upmu + {\mathbf{Zg}} + {\mathbf{x}}{\text{b}} + {\mathbf{e}} ,$$

where ${\mathbf{y}}_{{\mathbf{c}}}$, ${\mathbf{1}}$, $\upmu$, ${\mathbf{g}}$, ${\mathbf{Z}}$ and ${\mathbf{e}}$ are the same as in GBLUP, ${\text{b}}$ is the additive effect of the variant to be tested for association, and ${\mathbf{x}}$ is the vector of the variant’s genotype indicator variable coded as 0, 1 or 2. The analysis was based only on the reference data. Different p value cut-off levels (10^−1^ to 10^−7^) were used to assess the statistical significance of the effect of individual SNPs. When a SNP was determined to be significantly associated with corrected phenotypes based on the pre-specified significance cut-off level, the corresponding genomic region was considered to define a genomic feature.

Second, we derived genomic features from the summary statistics of a group of genetic markers located in a previously identified QTL region. All QTL for a specific trait were downloaded from NRSP (National Animal Genome Research Program), and gene annotation information was downloaded from Ensembl (http://www.ensembl.org). The markers were grouped according to the genomic locations of the QTL region for a specific trait category downloaded from the database. The genomic region spanned by each individual QTL was standardized to three bins of 100, 500 and 1000 kb on each side of the QTL midpoint. The SNPs that were mapped to the QTL region were considered to define a genomic feature.

### Evaluation of the accuracy of genomic prediction

The three methods, GBLUP, ssGBLUP, GFBLUP, were compared based on the accuracy of genomic predictions. The (ss)GBLUP models based on the 80K SNP panel, WGS data, and WGS data after LD pruning were termed (ss)GBLUP_80K, (ss)GBLUP_WGS, and (ss)GBLUP_LD, respectively. For GFBLUP, the genomic feature matrix was constructed based on prior information gained from QTL and GWAS strategies, and the results were termed GFBLUP_QTL and GFBLUP_GWAS, respectively.

To evaluate the accuracy of genomic prediction, the younger LM animals, based on birth dates after December 2013 for reproduction traits and after August 2014 for production traits, were selected as validation populations, with sizes of 223 and 270, respectively. Considering that, only reproduction records were available from the XD population and the small number of genotyped animals from the ZX population, younger XD animals (born after April 2013) were chosen as the validation population for reproduction traits, with a size of 196, which was similar to the size of the LM validation population. The accuracy of genomic prediction was evaluated as the correlation between GEBV and $y_{c}$ in the validation population. To evaluate the impact of using a combined reference population, the accuracy of genomic prediction based on the single and combined reference populations were compared for the production and reproduction traits.

## Results

### Population structure and genetic parameters

**Fig. 1:**
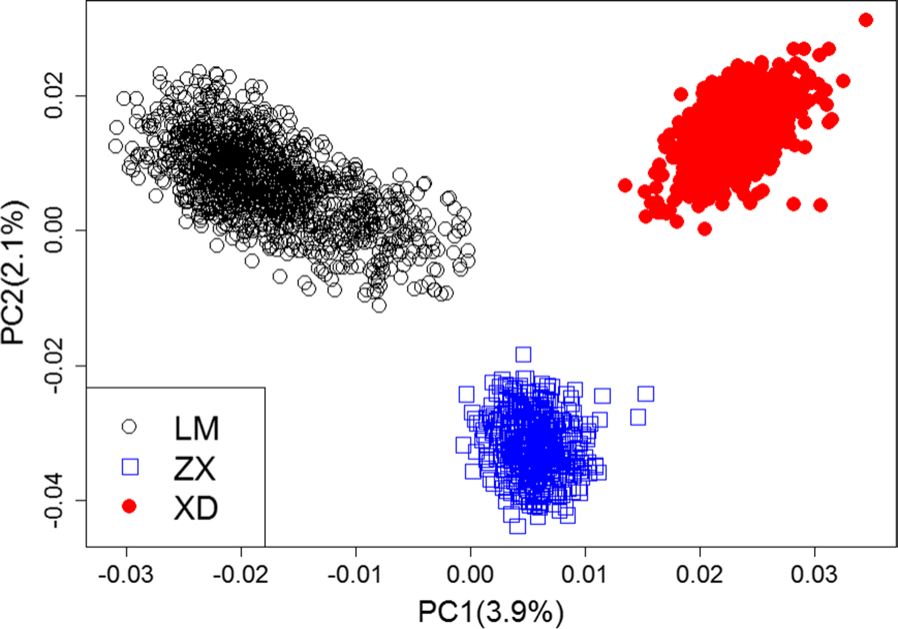
Principal component analysis (PCA) for three Yorkshire populations. XD, LM and ZX represent three Yorkshire populations from three Chinese pig breeding farms; PC1 (3.9%) = first principal component (variance explained by PC1 = 3.9%); PC2 (2.1%) = second principal component (variance explained by PC2 = 2.1%)

To identify the population structure of the three Yorkshire populations, principal component analysis (PCA) was performed using the 80K SNP panel. Figure 1 illustrates that the genetic backgrounds of the LM and ZX populations differed, although both were American Yorkshire progeny. Likewise, the XD population was distantly related to the LM and ZX groups due to its British origin.

Estimates of heritability of the reproduction and production traits in the three Yorkshire populations are in Table 1. Heritability estimates for the production traits ranged from 0.19 to 0.38 in the LM and ZX populations and for reproduction traits they were similar (0.07–0.09) in the LM and XD populations.

### Genotype imputation accuracy

**Fig. 2:**
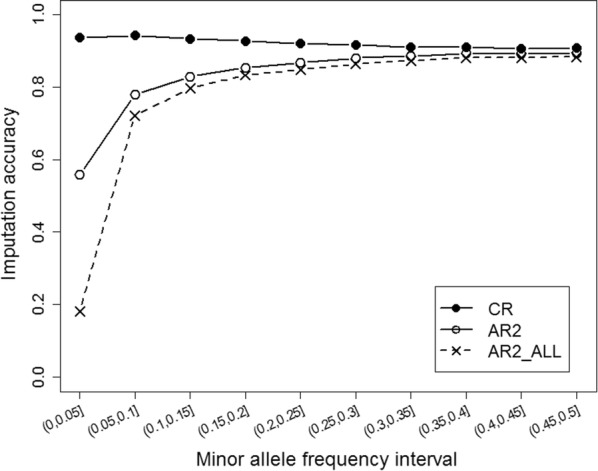
Imputation accuracy for each minor allele frequency (MAF) interval. Genotype concordance rate (CR), which was defined as the proportion of genotypes of the imputed variants that were the same as the whole-genome sequencing variants. AR2, allelic R-squared for consistent variants between imputation and whole-genome sequencing. AR2_ALL, allelic R-squared for all imputed variants

To assess the imputation accuracy for the imputed variants, we removed variants with minor allele frequencies (MAF) lower than 9.9E−5 and calculated the mean AR2 and mean CR across the range of MAF for the remaining variants, as shown in Fig. 2. The average CR across all variants was 0.92, which is sufficient for further analysis. The AR2 was sensitive to MAF. Variants with a MAF lower than 0.15 accounted for ~ 77% of the total number of variants, and the AR2 was extremely low for variants with a MAF lower than 0.05. For the variants with a MAF higher than 0.15, the AR2 was greater than 80%. Imputation accuracy was also investigated for each chromosome. As shown in Additional file 1: Figure S1 there were no significant differences between chromosomes.

### Genomic prediction accuracy

#### Comparison of the GBLUP method based on different marker densities

Genomic relationship matrices for use with GBLUP were constructed based on the different marker densities of the 80K SNP panel, the WGS data, and the WGS data after LD pruning. Table 2 presents the accuracy of genomic prediction for the reproduction traits based on the different reference populations and the LM validation population. For the LM reference population, prediction accuracies obtained with GBLUP_80K were 0.453 and 0.450 for NBA and TNB and were similar with GBLUP_WGS, i.e. 0.456 and 0.452, respectively. We found a similar trend in accuracies for the XD validation population, as shown in Table 3. For the XD reference population, prediction accuracies with GBLUP_80K and GBLUP_WGS were almost the same for NBA but slightly decreased for TNB from 0.431 with GBLUP_80K to 0.425 with GBLUP_WGS. Table 4 presents the accuracy of genomic prediction of the production traits for the LM validation population. There were no obvious differences in accuracy between GBLUP_80K and GBLUP_WGS for the LM reference population, e.g., the accuracy for AGE was 0.625 with GBLUP_80K and 0.633 with GBLUP_WGS.

**Table 2:** Accuracy of genomic prediction for reproduction traits using different reference populations to predict 223 younger individuals of the LM population

Reference set^a^ (size of reference population)	Method	Trait	
		NBA	TNB
LM (1418)	GBLUP_80K	0.453	0.450
	GBLUP_WGS	0.456	0.452
	GBLUP_LD	0.470	0.464
	GFBLUP_QTL	0.473	0.466
	GFBLUP_GWAS	0.464	0.459
	ssGBLUP_80K	0.649	0.664
	ssGBLUP_WGS	0.641	0.658
	ssGBLUP_LD	0.662	0.679
LM+XD (2180)	GBLUP_80K	0.459	0.460
	GBLUP_WGS	0.467	0.468
	GBLUP_LD	0.478	0.479
	GFBLUP_QTL	0.478	0.471
	GFBLUP_GWAS	0.462	0.455
	ssGBLUP_80K	0.646	0.664
	ssGBLUP_WGS	0.648	0.667
	ssGBLUP_LD	0.668	0.686
XD (762)	GBLUP_80K	0.026	− 0.016
	GBLUP_WGS	0.042	− 0.001
	GBLUP_LD	0.050	0.028
	GFBLUP_QTL	− 0.017	− 0.058
	GFBLUP_GWAS	− 0.008	− 0.009

*GBLUP_80K* genomic BLUP based on the 80K SNP panel; *GBLUP_WGS* GBLUP based on imputed whole-genome sequencing (WGS) data; *GBLUP_LD* GBLUP based on WGS after LD pruning; *GFBLUP_QTL* genomic feature BLUP with a genomic feature matrix constructed based on QTL prior information; *GFBLUP_GWAS* genomic feature BLUP with a genomic feature matrix constructed based on GWAS prior information; *ssGBLUP_80K, ssGBLUP_WGS, ssGBLUP_LD* For ssGBLUP, the H matrix was constructed based on the different genomic relationship matrices of the 80K chip panel, WGS data and WGS data after LD pruning and termed ssGBLUP_80K, ssGBLUP_WGS and ssGBLUP_LD, respectively; *NBA* number of piglets born alive; *TNB* total number of piglets born

^a^Yorkshire population LM, XD or LM plus the XD reference population

**Table 3:** Accuracy of genomic prediction for reproduction traits using different reference populations to predict 196 younger individuals of the XD population

Reference set^a^ (size of reference population)	Method	Trait	
		NBA	TNB
XD (566)	GBLUP_80K	0.392	0.431
	GBLUP_WGS	0.390	0.425
	GBLUP_LD	0.396	0.435
	GFBLUP_QTL	0.398	0.435
	GFBLUP_GWAS	0.378	0.425
	ssGBLUP_80K	0.441	0.465
	ssGBLUP_WGS	0.439	0.457
	ssGBLUP_LD	0.440	0.462
LM+XD (2207)	GBLUP_80K	0.387	0.439
	GBLUP_WGS	0.407	0.454
	GBLUP_LD	0.403	0.455
	GFBLUP_QTL	0.407	0.458
	GFBLUP_GWAS	0.429	0.494
	ssGBLUP_80K	0.451	0.480
	ssGBLUP_WGS	0.455	0.481
	ssGBLUP_LD	0.453	0.483
LM (1641)	GBLUP_80K	0.121	0.214
	GBLUP_WGS	0.204	0.275
	GBLUP_LD	0.152	0.228
	GFBLUP_QTL	0.163	0.232
	GFBLUP_GWAS	0.221	0.282

*GBLUP_80K* genomic BLUP based on the 80K SNP panel; *GBLUP_WGS* GBLUP based on imputed whole-genome sequencing (WGS) data; *GBLUP_LD* GBLUP based on WGS after LD pruning; *GFBLUP_QTL* genomic feature BLUP with a genomic feature matrix constructed based on QTL prior information; *GFBLUP_GWAS* genomic feature BLUP with a genomic feature matrix constructed based on GWAS prior information; *ssGBLUP_80K, ssGBLUP_WGS, ssGBLUP_LD* For ssGBLUP, the H matrix was constructed based on the different genomic relationship matrices of the 80K chip panel, WGS data and WGS data after LD pruning and termed ssGBLUP_80K, ssGBLUP_WGS and ssGBLUP_LD, respectively; *NBA* number of piglets born alive; *TNB* total number of piglets born

^a^Yorkshire population LM, XD or LM plus the XD reference population

**Table 4:** Accuracy of genomic prediction for production traits using different reference populations to predict 270 younger individuals of the LM population

Reference set^a^ (size of reference population)	Method	Trait	
		AGE	BFT
LM (1499)	GBLUP_80K	0.625	0.432
	GBLUP_WGS	0.633	0.436
	GBLUP_LD	0.642	0.445
	GFBLUP_QTL	0.642	0.444
	GFBLUP_GWAS	0.640	0.444
	ssGBLUP_80K	0.739	0.677
	ssGBLUP_WGS	0.738	0.663
	ssGBLUP_LD	0.745	0.671
LM+ZX (1819)	GBLUP_80K	0.615	0.431
	GBLUP_WGS	0.636	0.443
	GBLUP_LD	0.648	0.454
	GFBLUP_QTL	0.638	0.443
	GFBLUP_GWAS	0.635	0.452
	ssGBLUP_80K	0.737	0.677
	ssGBLUP_WGS	0.739	0.664
	ssGBLUP_LD	0.746	0.673
ZX (320)	GBLUP_80K	0.028	0.067
	GBLUP_WGS	0.098	0.111
	GBLUP_LD	0.164	0.142
	GFBLUP_QTL	0.164	0.144
	GFBLUP_GWAS	0.124	0.119

*GBLUP_80K* genomic BLUP based on the 80K SNP panel; *GBLUP_WGS* GBLUP based on imputed whole-genome sequencing (WGS) data; *GBLUP_LD* GBLUP based on WGS after LD pruning; *GFBLUP_QTL* genomic feature BLUP with a genomic feature matrix constructed based on QTL prior information; *GFBLUP_GWAS* genomic feature BLUP with a genomic feature matrix constructed based on GWAS prior information; *ssGBLUP_80K, ssGBLUP_WGS, ssGBLUP_LD* For ssGBLUP, the H matrix was constructed based on the different genomic relationship matrices of the 80K chip panel, WGS data and WGS data after LD pruning and termed ssGBLUP_80K, ssGBLUP_WGS and ssGBLUP_LD, respectively; *AGE* days to 100 kg; *BFT* backfat thickness

^a^Yorkshire population of LM, ZX or LM plus the ZX reference population

It should be noted that, compared to GBLUP_80K and GBLUP_WGS, GBLUP_LD resulted in a higher accuracy for all scenarios (Tables 2, 3, 4); improvements were on average 1.4 and 1.0% for reproduction traits and 2.2 and 1.0% for production traits, respectively. The highest increase was 3.3% for AGE with GBLUP_LD compared to GBLUP_80K for the LM+ZX reference population and the LM validation population. This result indicates that pruning SNPs that are in complete or high LD with other SNPs is an effective strategy to reduce the number of uninformative markers and increase the prediction accuracy with WGS data.

#### Accuracy of GFBLUP methods with preselection and prior biological information

To evaluate the effect of including prior information, GFBLUP methods with preselection and prior biology strategies were compared with GBLUP_WGS and other methods. Prediction accuracies differed for different QTL lengths or GWAS p values used for GFBLUP (see Additional file 2: Table S1, Additional file 3: Table S2, Additional file 4: Table S3, Additional file 5: Table S4, Additional file 6: Table S5, Additional file 7: Table S6, Additional file 8: Table S7, Additional file 9: Tables S8]. The best results for GFBLUP are in Tables 2, 3 and 4. In the scenario in which a single population was used as the reference population, the accuracy of genomic prediction increased by on average 1.1 and 1.5% for GFBLUP_QTL and GFBLUP_GWAS, respectively, compared to GBLUP_WGS (Tables 2, 3 and 4), although in some scenarios, accuracy did not increase or slightly decreased, e.g., with the XD reference and validation population for NBA, prediction accuracies were 0.390 and 0.378 for GBLUP_WGS and GFBLUP_GWAS, respectively (Table 3). Therefore, preselection of variants that might be causal on the basis of prior biological knowledge (e.g., Gene Ontology and pathway) may be key to improving prediction accuracy.

#### Accuracy of ssGBLUP

In this study, the genomic relationship matrix ${\mathbf{H}}$ for ssGBLUP was based on the same marker densities as those used for GBLUP. Among the three approaches (GBLUP, GFBLUP and ssGBLUP) used to predict genomic breeding values, ssGBLUP performed best. For the LM validation population in Table 2, the accuracy of prediction obtained by ssGBLUP ranged from 0.641 to 0.686 for NBA and TNB. Averaged across the two traits, ssGBLUP yielded a 20.1% higher accuracy than GBLUP and a 19.3% higher accuracy than GFBLUP for a given scenario. A similar trend was also found for the XD validation population for reproduction traits and for the LM validation population for production traits (Tables 3 and 4). As shown in Table 3, on average, ssGBLUP had a 4.1 and 3.1% higher accuracy than GBLUP and GFBLUP, respectively. The size of the increase in accuracy from using ssGBLUP was small, which could be due to the small number of genotyped animals (the XD reference population included 556 animals). In addition, with the XD reference population, accuracy was on average slightly higher for GFBLUP_GWAS (0.494) than for ssGBLUP (0.481) with the LM+XD reference population (Table 3). Table 4 presents the accuracy of genomic prediction for production traits with ssGBLUP. Among the three genomic prediction methods used, ssGBLUP on average yielded a 17.0 and 16.4% higher accuracy than GBLUP and GFBLUP, respectively.

The accuracies of prediction obtained with ssGBLUP when different marker densities were used for the construction of ${\mathbf{H}}$ were also compared, as shown in Tables 2, 3 and 4. For most scenarios, ssGBLUP_LD performed better than ssGBLUP_80K and ssGBLUP_WGS for both the reproduction and production traits. However, ssGBLUP_LD performed almost as well as ssGBLUP_80K and ssGBLUP_WGS with the XD validation population (Table 3). Furthermore, in the scenarios that used the LM and LM+ZX reference populations, the accuracy of ssGBLUP_LD was slightly lower than that of ssGBLUP_80K for the production traits (Table 4).

#### Impact of the combined reference population on accuracy of genomic prediction

Our main objective was to investigate how much the accuracy of genomic prediction can increase by using combined populations with different genetic backgrounds and WGS data instead of lower-density marker panels. For the reproduction traits with the LM validation population (Table 2), the accuracy of genomic predictions based on GBLUP_80K increased slightly from 0.453 and 0.450 to 0.459 and 0.460 for NBA and TNB, respectively, when the reference population was enlarged from only the LM population to the admixed population LM+XD. Corresponding average increases in accuracy were 1.4 and 1.2% for GBLUP_WGS and GBLUP_LD, respectively. For the same scenarios, the accuracy of prediction was not improved or decreased slightly when combining the reference populations for ssGBLUP_80K for NBA and TNB, while both GBLUP_WGS and GBLUP_LD yielded approximately 1% higher accuracy with the combined reference population. For the XD validation population (Table 3), prediction accuracy for NBA and TNB decreased or slightly increased for GBLUP with the combined reference population based on the low-density marker panel. However, when WGS data were used, prediction accuracy on average improved by 2.3 and 1.4% for NBA and TNB with GBLUP_WGS and GBLUP_LD, respectively. For ssGBLUP, average prediction accuracy for NBA and TNB increased when combining reference populations, by 1.3, 2, and 1.7% for ssGBLUP_80 K, ssGBLUP_WGS, and ssGBLUP_LD, respectively. The improvement in prediction accuracy from combining reference populations was greater for the WGS data than for the 80K SNP panel.

For production traits, prediction accuracy for AGE and BFT based on the combined reference population LM+ZX compared to the single reference population LM decreased for GBLUP based on the 80K SNP panel (Table 4). However, when WGS data were used, prediction accuracy increased, although the increase was small, at approximately 1%, for GBLUP_WGS and GBLUP_LD. A similar trend was also found for ssGBLUP, where a small advantage was obtained when WGS data were used compared to the 80K marker panel. The reason for the small advantage of the combined reference population for production traits may be because the LM population was already large, and the small size of the ZX population did not provide much additional information.

Tables 2, 3 and 4 show that there was no advantage from using the combined population genomic prediction for the GFBLUP method. For the reproduction traits with the LM validation population, as shown in Table 2, similar prediction accuracies were obtained with the single and the combined reference population. However, for the XD validation population (see Table 3), the accuracy of genomic prediction with GFBLUP increased for reproduction traits when using the combined LM + XD reference population compared to using the XD population alone. For the production traits (see Table 4), the accuracy of genomic prediction decreased slightly with the combined reference population, except that the accuracy for BFT increased from 0.444 to 0.452 with GFBLUP_GWAS.

We also performed genomic prediction across populations with different genetic backgrounds. When the XD population was used for genomic prediction of reproduction traits in LM (see Table 2), i.e. when no animals from the LM population were included in the reference population, prediction accuracies obtained with GBLUP_80K were as low as 0.026 and − 0.016 for NBA and TNB, respectively. However, GBLUP_WGS and GBLUP_LD yielded a 1.6 (1.5) and 2.4% (4.4%) higher accuracy, respectively, for this scenario than GBLUP_80K for NBA (TNB), while GFBLUP yielded a lower accuracy than GBLUP_80K. Likewise, when using LM to predict XD, the accuracy of prediction obtained with GBLUP_80K was 0.121 and 0.214 for NBA and TNB, respectively, and increased with GBLUP_WGS and GBLUP_LD. For the production traits (see Table 4), when using ZX to predict LM, a higher prediction accuracy was also obtained for all scenarios when WGS data were used than when the 80K SNP panel was used, which further supports the advantage of using WGS data in combined population genomic prediction.

## Discussion

### Impact of marker density on accuracy of genomic prediction

In this study, we investigated the accuracy of genomic prediction based on imputed WGS data versus a medium-density SNP panel using real pig data. In theory, using WGS data in genomic prediction is expected to lead to higher predictive ability, because WGS data include most of the causal mutations that influence traits of interest, and prediction is much less limited by LD between SNPs and causal mutations [32, 33]. In addition, simulation studies suggested that WGS data could improve the accuracy of genomic prediction within a population by as much as 40%, depending on the trait, statistical method, and MAF of the causal mutations affecting the traits [17, 34]. However, we observed no increase in accuracy when imputed sequence data was used for within-population prediction compared to 50K or 80K SNP data. An example is the genomic prediction of reproduction and production traits when both the reference and validation populations were from LM, (see Tables 2 and 4), and we observed an even lower accuracy with imputed sequence data than with the 80K SNP chip for TNB within the XD population (see Table 3). Similar results were reported for feed efficiency component traits in Duroc pigs [8], backfat thickness in pigs [35], and body conformation traits in Chinese Holsteins using imputed HD data [36].

When using imputed WGS data for genomic prediction, several factors can affect accuracy of resulting predictions:

1. Genotype imputation accuracy. In the current study, we obtained a high average imputation accuracy of 0.92 from 80K to WGS. However, potential imputation errors are difficult to detect, which affects the accuracy of genomic prediction. van Binsbergen et al. [7] reported that the predictive accuracy was lower with imputed HD array data than with genotyped HD array data for a population of more than 5000 Holstein-Friesian cattle. Currently, sequencing all individuals in a population is not realistic. Thus, to benefit from the advantage of using WGS data instead of high-density genotype data for genomic prediction, it is necessary to target a large training set with a small average relationship between animals to increase imputation accuracy [37].
2. LD pruning of WGS data. One basic assumption of GS is that each QTL is in LD with at least one SNP; thus, SNPs that are distributed across the whole genome can explain most of the genetic variance [1]. However, when two SNPs are in high LD, their genotypic information is redundant and only one is necessary to represent the variation in neighboring regions, especially for WGS data. Too many SNPs with high LD may introduce noise and result in biased genomic prediction. Based on our results, pruning SNPs that are in complete or high LD with other SNPs is an effective strategy to improve the accuracy of genomic prediction, as higher accuracy was obtained with GBLUP and ssGBLUP for almost all the scenarios (Tables 2, 3 and 4) when high LD SNPs ($r^{2}$ > 0.9) were removed from the WGS data. Removing uninformative SNPs also decreased the computational demand for the construction of the ${\mathbf{G}}$ matrix. Similar results were obtained in Holstein-Friesian bulls with an LD threshold for pruning of 0.9 [22].
3. Preselection and prior biology of sequence variants. WGS data are expected to capture genetic variation more completely than SNP panels, but the direct use of WGS data did not yield an advantage in our study. Many studies have demonstrated that adding significant markers derived from HD marker panels or sequence data into medium (50K or 80K) density panel data can improve the accuracy of genomic prediction [9, 36, 38], since it treats significant SNPs as genomic features (as in GFBLUP, which is discussed below). However, in this study, accuracy did not increase by adding preselected GWAS markers from the WGS data to the 80K SNP panel (results not shown), which agrees with the results of Veerkamp et al. [39] and Calus et al. [22]. The lack of improvement in accuracy may be due to the genetic architecture of the trait or the limited ability to correctly estimate QTL.

### Comparison of methods of genomic prediction

In this study, three different methods, GBLUP, ssGBLUP and GFBLUP, were compared. To date, the use of GFBLUP and ssGBLUP with WGS data in pigs has rarely been investigated. The ssGBLUP model uses a combined genotype-pedigree relationship matrix [24, 25], and in this study, all ungenotyped animals were added to construct the ${\mathbf{H}}$ matrix. As expected, ssGBLUP performed better than GBLUP and GFBLUP for all scenarios, which is consistent with other reports [6, 12]. Our results show that ssGBLUP did not yield a higher accuracy of genomic prediction with WGS data than with the 80K SNP panel in the same scenarios. The possible reason is that the ${\mathbf{A}}$ matrix portion of the construction of the ${\mathbf{H}}$ matrix was the same, and that the other part of the genomic relationship matrix was based on the 80K SNP panel and WGS, whereas increasing the number of markers used for prediction to the WGS level does not increase the accuracy, as discussed above.

In this study, we investigated the efficiency of GFBLUP based on two sources of additional information: incorporating prior knowledge of QTL from the literature (GFBLUP_QTL) and including significant SNPs obtained from GWAS (GFBLUP_GWAS) as known genomic features. Our results indicate that GFBLUP yielded approximately 1 to 2% higher accuracy than GBLUP based on the WGS data for the reproduction and production traits (see Tables 2, 3 and 4). This is consistent with other studies [29, 40]. Fang et al. [40] reported that the accuracy of genomic prediction was marginally improved (approximately 3%) with GFBLUP compared to standard GBLUP when using imputed sequence variants in Holstein and Jersey cattle. The advantage of GFBLUP over GBLUP is attributed mainly to the fact that GFBLUP allows the assignment of different weights to the genomic variants in the different genomic relationship based on their estimated genomic parameters, which can better fit the genetic architecture of the trait [29, 40]. However, GFBLUP was not superior to GBLUP for all scenarios, e.g., for prediction within the XD population, prediction accuracies for NBA were 0.390 and 0.378 with GBLUP_WGS and GFBLUP_GWAS, respectively (Table 3). The imperfect imputation of WGS variants may be a factor that limits the predictive ability of GFBLUP, as noted in other studies [29, 41].

Genomic features based on published QTL includes non-causal markers, and the genetic architecture of complex traits is currently poorly revealed by GWAS, owing to the many individually undetectable QTL with small to moderate effects [42], which could affect the predictive ability of GFBLUP. Furthermore, the model may be trait-specific, e.g., the total heritability of a trait and the number of markers per genomic feature differ by trait, which can cause variation in the accuracy of genomic prediction (see Additional files 2 to 9: Tables S1 to S8). Moreover, published QTL are not available for some traits (e.g., farrowing interval of sows), and prior QTL information is far from complete for most traits of interest. All these factors limit the use of GFBLUP with publicly available QTL data. The number of SNPs in genomic features obtained by using QTLdb or GWAS affects the estimate of the genomic feature variance, as shown in Additional files 2 to 9: Tables S1 to S8. The prediction accuracy of GFBLUP is influenced both by the genomic variance explained by the genomic features and by the number of noisy SNPs that are present in each feature. In addition, any errors in estimates of variance components will reduce prediction accuracy, because using incorrect variance components results in the genomic relationships not being used for prediction in an optimal manner [43].

However, our results indicated that GFBLUP had no advantage for genomic prediction in the combined population compared to single-population genomic prediction, e.g., as shown in Table 2, for the LM validation population, the accuracy of GS for TNB with GFBLUP_GWAS decreased from 0.459 for the LM reference population to 0.455 for the combined LM+XD reference population. Furthermore, GFBLUP_QTL did not increase accuracy of prediction for the combined LM+XD reference population compared to the LM reference population, which could be caused by non-persistent associations between SNPs and QTL or inconsistent LD patterns between SNPs and QTL across populations. Thus, the same genomic feature matrix in a single population for GFBLUP should not be used for the combined population.

### Combined-population genomic prediction based on WGS data

Reference population size and relationships between the reference and validation populations are key factors for the accuracy of genomic prediction. Generally, the accuracy of genomic prediction increases with increasing reference population size [44]. However, assembling a large reference population is challenging due to the relatively small population sizes of some breeds or farms. Combining populations into one common reference population has been very useful for genomic prediction, e.g., in dairy and beef cattle [2, 3, 13, 14]. In this study, we assessed the advantage of a combined reference population for genomic prediction. Our previous investigation showed that the combined reference population in pigs did not have an advantage over a single population in all scenarios and even performed more poorly with the 80K SNP panel [6]. This was confirmed by our findings in this study, which enlarged the genotyped population compared to our previous investigation (Tables 2, 3 and 4). This phenomenon was also reported in other studies [4, 15, 45] and can be due to three factors: (1) inconsistent LD between SNPs and QTL across populations in the SNP-chip panel [15, 44]; (2) an increase in the genetic distance between individuals of the reference and validation populations, resulting in lower accuracy for the combined reference population [46]; and (3) differences in allele substitution effects between populations, resulting in differences in the components of that variance in terms of the contribution of each QTL, which could impact prediction in the combined population [47].

However, our results also indicated that the accuracy of genomic prediction increased by 1 to 2.4% and by 1% when using WGS data instead of the 80K SNP panel with GBLUP and ssGBLUP, respectively, in all scenarios (Tables 2, 3 and 4). Iheshiulor et al. [17] also reported that the use of WGS data was especially beneficial for multibreed prediction. This improvement is mainly due to improvements in the three factors discussed above. For example, when calculating the ${\mathbf{G}}$-matrix based on the LM and XD populations, the number of genomic relationships between the two populations that are greater than 0.1 increased from 741 for the 80K SNP panel to 21,310 for the WGS data. Hayes et al. [33] also noted that the main reason for the benefit of WGS data in combined populations and across populations was that the presence of QTL in the WGS data increased the probability of picking up similar QTL that segregate between populations.

However, the size of the increase in accuracy from using WGS was still small. Several other sources of improvement could be explored: (1) incorporating imputation accuracy into genomic prediction models by weighting all SNPs with imputation accuracy of AR2 for the construction of the ${\mathbf{G}}$ matrix; (2) using two Bayesian methods of split-and-merge Bayesian variable selection [22] and BayesR to drop variants with a small effect [48] for the combined population prediction to fit the model and reduce the computing time, since the Bayesian method can theoretically capture all the variants provided by the WGS data; (3) using an LD-adjusted kinship matrix instead of a standard kinship matrix in genomic prediction models to eliminate the biases due to overestimation in regions of strong LD and underestimation in regions of low LD, as described by Speed et al. [49]; and (4) using a multitrait model for genomic prediction in multipopulation reference populations, in which the same trait in different populations is considered as a different trait. For populations with similar genetic backgrounds, i.e., for which genetic relationships between populations are sufficient, another advantage of using a multitrait approach is that it accounts for potential genotype-by-environment interactions (G × E) to improve the accuracy of genomic prediction [50, 51]. For multitrait GBLUP, a multipopulation genomic relationship matrix can be used to account for the genetic relationships between populations [52]. However, it might not be possible to apply multitrait ssGBLUP if there is little or no pedigree relationship between populations. In addition, the computational demand of the multitrait approach will increase rapidly with an increase in the number of populations, as more (co)variance components will have to be estimated, and it will be more difficult to converge. However, the efficiency of these approaches in actual genome prediction requires further investigation.

## Conclusions

The use of WGS data holds potential to increase the accuracy of combined-population genomic prediction, and ssGBLUP performed best in all scenarios. However, WGS is still much more expensive than SNP-chip assays; thus, a more acceptable approach is to sequence a subset of a population as a reference panel to perform genotype imputation with high accuracy. Our results showed that simply increasing the number of markers used for prediction to the WGS level does not increase the accuracy of single-population prediction, while pruning WGS data and using GFBLUP based on prior information could yield higher accuracy than prediction based on a SNP panel.

## Supplementary information

**Additional file 1: Figure S1.** Imputation accuracy across chromosomes.

**Additional file 2: Tables S1.** Accuracy of genomic prediction for days to 100 kg (AGE) with different QTL lengths as prior information in GFBLUP.

**Additional file 3: Tables S2.** Accuracy of genomic prediction for backfat thickness (BFT) with different QTL lengths as prior information in GFBLUP.

**Additional file 4: Tables S3.** Accuracy of genomic prediction for number of piglets born alive (NBA) with different QTL lengths as prior information in GFBLUP.

**Additional file 5: Tables S4.** Accuracy of genomic prediction for total number of piglets born (TNB) with different QTL lengths as prior information in GFBLUP.

**Additional file 6: Tables S5.** Accuracy of genomic prediction for days to 100 kg (AGE) with different p values of GWAS as prior information in GFBLUP.

**Additional file 7: Tables S6.** Accuracy of genomic prediction for backfat thickness (BFT) with different p values of GWAS as prior information in GFBLUP.

**Additional file 8: Tables S7.** Accuracy of genomic prediction for number of piglets born alive (NBA) with different p values of GWAS as prior information in GFBLUP.

**Additional file 9: Tables S8.** Accuracy of genomic prediction for total number of piglets born (TNB) with different p values of GWAS as prior information in GFBLUP.

## Data Availability

The datasets used during the current study are available from the corresponding author on reasonable request.
