# Supplementary material for: Using imputation-based whole-genome sequencing data to improve the accuracy of genomic prediction for combined populations in pigs
Source: Genet Sel Evol. 2019 Oct 21;51:58. doi: 10.1186/s12711-019-0500-8 (PMC6805481; doi:10.1186/s12711-019-0500-8)
Supplement: Supplementary file 1 — Additional file 1: Figure S1. Imputation accuracy across chromosomes. [file 12711_2019_500_MOESM1_ESM.docx]

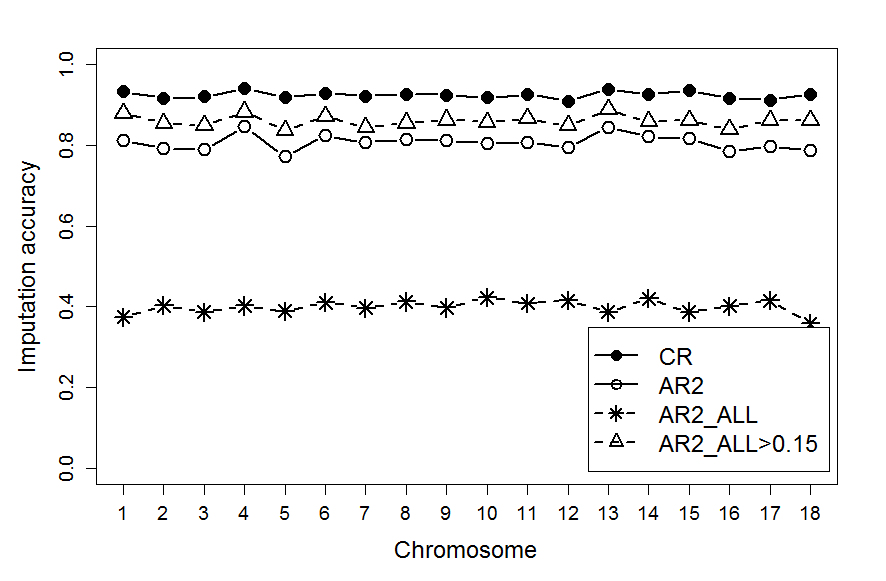


**Figure S1.** Imputation accuracy across chromosome. CR, genotype concordance rate which was defined as the proportion of genotypes of imputed variants, which were the same as the whole genome sequencing. AR2, allelic R-squared for consistent variants between imputation and whole genome sequencing. AR2_ALL, allelic R-squared for all imputed variants. AR2_ALL>0.15, allelic R-squared for all imputed variants with MAF>0.15.
