# Supplementary material for: Using imputation-based whole-genome sequencing data to improve the accuracy of genomic prediction for combined populations in pigs
Source: Genet Sel Evol. 2019 Oct 21;51:58. doi: 10.1186/s12711-019-0500-8 (PMC6805481; doi:10.1186/s12711-019-0500-8)
Supplement: Supplementary file 2 — Additional file 2: Tables S1. Accuracy of genomic prediction for days to 100 kg (AGE) with different QTL lengths as prior information in GFBLUP. [file 12711_2019_500_MOESM2_ESM.docx]

**Table S1.** The accuracy of genomic prediction for days to 100 kg (AGE) with different QTL lengths as prior information on GFBLUP.

| QTL length(kb) | SNP number | ref | tar | cor | varA | varE | varR | $h^{2}$ |
| --- | --- | --- | --- | --- | --- | --- | --- | --- |
| 100 | 4 | LM+ZX | LM | 0.633 | 119.780 | 3.408 | 42.857 | 0.742 |
| 500 | 1217 | LM+ZX | LM | 0.638 | 120.153 | 0.000 | 43.046 | 0.736 |
| 1000 | 1217 | LM+ZX | LM | 0.638 | 120.153 | 0.000 | 43.046 | 0.736 |
| 100 | 4 | ZX | LM | 0.164 | 44.916 | 0.000 | 19.678 | 0.695 |
| 500 | 1217 | ZX | LM | 0.155 | 43.111 | 2.603 | 20.102 | 0.695 |
| 1000 | 1217 | ZX | LM | 0.155 | 43.111 | 2.603 | 20.102 | 0.695 |
| 100 | 4 | LM | LM | 0.637 | 136.726 | 4.268 | 44.720 | 0.759 |
| 500 | 1217 | LM | LM | 0.642 | 137.728 | 0.000 | 44.817 | 0.754 |
| 1000 | 1217 | LM | LM | 0.642 | 137.728 | 0.000 | 44.817 | 0.754 |

ref: reference population; tar: validation population;

cor: accuracy of genomic prediction;

varA: variance components accounted for by the remaining genome;

varE: variance components accounted for by the variants in the genomic feature;

varR: residual variance component.
