# Supplementary material for: Using imputation-based whole-genome sequencing data to improve the accuracy of genomic prediction for combined populations in pigs
Source: Genet Sel Evol. 2019 Oct 21;51:58. doi: 10.1186/s12711-019-0500-8 (PMC6805481; doi:10.1186/s12711-019-0500-8)
Supplement: Supplementary file 3 — Additional file 3: Tables S2. Accuracy of genomic prediction for backfat thickness (BFT) with different QTL lengths as prior information in GFBLUP. [file 12711_2019_500_MOESM3_ESM.docx]

**Table S2.** The accuracy of genomic prediction for backfat thickness (BFT) with different QTL lengths as prior information on GFBLUP.

| QTL length(kb) | SNP number | ref | tar | cor | varA | varE | varR | $h^{2}$ |
| --- | --- | --- | --- | --- | --- | --- | --- | --- |
| 100 | 2738 | LM+ZX | LM | 0.443 | 3.592 | 0.083 | 1.690 | 0.685 |
| 500 | 26981 | LM+ZX | LM | 0.442 | 3.360 | 0.304 | 1.697 | 0.683 |
| 1000 | 81092 | LM+ZX | LM | 0.443 | 3.678 | 0.000 | 1.688 | 0.685 |
| 100 | 2738 | ZX | LM | 0.142 | 1.634 | 0.000 | 0.391 | 0.807 |
| 500 | 26981 | ZX | LM | 0.144 | 1.631 | 0.003 | 0.392 | 0.807 |
| 1000 | 81092 | ZX | LM | 0.137 | 1.637 | 0.000 | 0.391 | 0.807 |
| 100 | 2738 | LM | LM | 0.444 | 4.146 | 0.178 | 1.794 | 0.707 |
| 500 | 26981 | LM | LM | 0.444 | 4.087 | 0.199 | 1.801 | 0.704 |
| 1000 | 81092 | LM | LM | 0.443 | 4.322 | 0.000 | 1.790 | 0.707 |

ref: reference population; tar: validation population;

cor: accuracy of genomic prediction;

varA: variance components accounted for by the remaining genome;

varE: variance components accounted for by the variants in the genomic feature;

varR: residual variance component.
