# Supplementary material for: Using imputation-based whole-genome sequencing data to improve the accuracy of genomic prediction for combined populations in pigs
Source: Genet Sel Evol. 2019 Oct 21;51:58. doi: 10.1186/s12711-019-0500-8 (PMC6805481; doi:10.1186/s12711-019-0500-8)
Supplement: Supplementary file 4 — Additional file 4: Tables S3. Accuracy of genomic prediction for number of piglets born alive (NBA) with different QTL lengths as prior information in GFBLUP. [file 12711_2019_500_MOESM4_ESM.docx]

**Table S3.** The accuracy of genomic prediction for number of piglets born alive (NBA) with different QTL lengths as prior information on GFBLUP.

| QTL length(kb) | SNP number | ref | tar | cor | varA | varE | varR | $h^{2}$ |
| --- | --- | --- | --- | --- | --- | --- | --- | --- |
| 100 | 1545 | LM+XD | LM | 0.478 | 1.198 | 0.039 | 0.457 | 0.730 |
| 500 | 15714 | LM+XD | LM | 0.472 | 1.233 | 0.000 | 0.455 | 0.730 |
| 1000 | 21017 | LM+XD | LM | 0.472 | 1.234 | 0.000 | 0.455 | 0.731 |
| 100 | 1545 | LM+XD | XD | 0.407 | 1.198 | 0.039 | 0.457 | 0.730 |
| 500 | 15714 | LM+XD | XD | 0.403 | 1.233 | 0.000 | 0.455 | 0.730 |
| 1000 | 21017 | LM+XD | XD | 0.403 | 1.234 | 0.000 | 0.455 | 0.731 |
| 100 | 1545 | XD | LM | -0.018 | 0.598 | 0.000 | 0.227 | 0.725 |
| 500 | 15714 | XD | LM | -0.022 | 0.584 | 0.017 | 0.227 | 0.726 |
| 1000 | 21017 | XD | LM | -0.017 | 0.598 | 0.000 | 0.227 | 0.725 |
| 100 | 1545 | XD | XD | 0.396 | 0.598 | 0.000 | 0.227 | 0.725 |
| 500 | 15714 | XD | XD | 0.398 | 0.584 | 0.017 | 0.227 | 0.726 |
| 1000 | 21017 | XD | XD | 0.395 | 0.598 | 0.000 | 0.227 | 0.725 |
| 100 | 1545 | LM | LM | 0.473 | 1.578 | 0.016 | 0.502 | 0.761 |
| 500 | 15714 | LM | LM | 0.470 | 1.598 | 0.000 | 0.500 | 0.762 |
| 1000 | 21017 | LM | LM | 0.470 | 1.600 | 0.000 | 0.500 | 0.762 |
| 100 | 1545 | LM | XD | 0.163 | 1.578 | 0.016 | 0.502 | 0.761 |
| 500 | 15714 | LM | XD | 0.160 | 1.598 | 0.000 | 0.500 | 0.762 |
| 1000 | 21017 | LM | XD | 0.160 | 1.600 | 0.000 | 0.500 | 0.762 |

ref: reference population; tar: validation population;

cor: accuracy of genomic prediction;

varA: variance components accounted for by the remaining genome;

varE: variance components accounted for by the variants in the genomic feature;

varR: residual variance component.
