# Supplementary material for: Using imputation-based whole-genome sequencing data to improve the accuracy of genomic prediction for combined populations in pigs
Source: Genet Sel Evol. 2019 Oct 21;51:58. doi: 10.1186/s12711-019-0500-8 (PMC6805481; doi:10.1186/s12711-019-0500-8)
Supplement: Supplementary file 5 — Additional file 5: Tables S4. Accuracy of genomic prediction for total number of piglets born (TNB) with different QTL lengths as prior information in GFBLUP. [file 12711_2019_500_MOESM5_ESM.docx]

**Table S4.** The accuracy of genomic prediction for total number of piglets born (TNB) with different QTL lengths as prior information on GFBLUP.

| QTL length(kb) | SNP number | ref | tar | cor | varA | varE | varR | $h^{2}$ |
| --- | --- | --- | --- | --- | --- | --- | --- | --- |
| 100 | 448 | LM+XD | LM | 0.471 | 1.662 | 0.042 | 0.579 | 0.746 |
| 500 | 14976 | LM+XD | LM | 0.467 | 1.703 | 0.000 | 0.577 | 0.747 |
| 1000 | 41358 | LM+XD | LM | 0.467 | 1.702 | 0.000 | 0.577 | 0.747 |
| 100 | 448 | LM+XD | XD | 0.458 | 1.662 | 0.042 | 0.579 | 0.746 |
| 500 | 14976 | LM+XD | XD | 0.451 | 1.703 | 0.000 | 0.577 | 0.747 |
| 1000 | 41358 | LM+XD | XD | 0.452 | 1.702 | 0.000 | 0.577 | 0.747 |
| 100 | 448 | XD | LM | -0.058 | 0.807 | 0.000 | 0.214 | 0.790 |
| 500 | 14976 | XD | LM | -0.072 | 0.775 | 0.041 | 0.212 | 0.794 |
| 1000 | 41358 | XD | LM | -0.070 | 0.725 | 0.097 | 0.212 | 0.795 |
| 100 | 448 | XD | XD | 0.435 | 0.807 | 0.000 | 0.214 | 0.790 |
| 500 | 14976 | XD | XD | 0.433 | 0.775 | 0.041 | 0.212 | 0.794 |
| 1000 | 41358 | XD | XD | 0.431 | 0.725 | 0.097 | 0.212 | 0.795 |
| 100 | 448 | LM | LM | 0.466 | 2.214 | 0.012 | 0.656 | 0.772 |
| 500 | 14976 | LM | LM | 0.462 | 2.232 | 0.000 | 0.654 | 0.773 |
| 1000 | 41358 | LM | LM | 0.463 | 2.232 | 0.000 | 0.653 | 0.774 |
| 100 | 448 | LM | XD | 0.232 | 2.214 | 0.012 | 0.656 | 0.772 |
| 500 | 14976 | LM | XD | 0.224 | 2.232 | 0.000 | 0.654 | 0.773 |
| 1000 | 41358 | LM | XD | 0.224 | 2.232 | 0.000 | 0.653 | 0.774 |

ref: reference population; tar: validation population;

cor: accuracy of genomic prediction;

varA: variance components accounted for by the remaining genome;

varE: variance components accounted for by the variants in the genomic feature;

varR: residual variance component.
