# Supplementary material for: Using imputation-based whole-genome sequencing data to improve the accuracy of genomic prediction for combined populations in pigs
Source: Genet Sel Evol. 2019 Oct 21;51:58. doi: 10.1186/s12711-019-0500-8 (PMC6805481; doi:10.1186/s12711-019-0500-8)
Supplement: Supplementary file 6 — Additional file 6: Tables S5. Accuracy of genomic prediction for days to 100 kg (AGE) with different p values of GWAS as prior information in GFBLUP. [file 12711_2019_500_MOESM6_ESM.docx]

**Table S5.** The accuracy of genomic prediction for days to 100 kg (AGE) with different p values of GWAS as prior information on GFBLUP.

| p-value | SNP number | ref | tar | cor | varA | varE | varR | $h^{2}$ |
| --- | --- | --- | --- | --- | --- | --- | --- | --- |
| 10^-1^ | 1408517 | LM+ZX | LM | 0.635 | 0.000 | 90.776 | 40.971 | 0.689 |
| 10^-2^ | 632538 | LM+ZX | LM | 0.634 | 0.000 | 78.055 | 42.158 | 0.649 |
| 10^-3^ | 302932 | LM+ZX | LM | 0.633 | 0.000 | 73.053 | 43.607 | 0.626 |
| 10^-4^ | 148881 | LM+ZX | LM | 0.625 | 0.000 | 75.812 | 45.170 | 0.627 |
| 10^-5^ | 75354 | LM+ZX | LM | 0.624 | 3.414 | 80.803 | 46.315 | 0.645 |
| 10^-6^ | 39032 | LM+ZX | LM | 0.619 | 10.867 | 85.358 | 46.946 | 0.672 |
| 10^-7^ | 20529 | LM+ZX | LM | 0.609 | 17.455 | 96.997 | 47.786 | 0.705 |
| 10^-8^ | 10834 | LM+ZX | LM | 0.601 | 23.187 | 112.684 | 48.352 | 0.738 |
| 10^-9^ | 5864 | LM+ZX | LM | 0.593 | 27.407 | 156.559 | 48.909 | 0.790 |
| 10^-1^ | 917617 | ZX | LM | 0.124 | 0.000 | 36.495 | 15.701 | 0.699 |
| 10^-2^ | 262020 | ZX | LM | 0.059 | 0.000 | 36.190 | 15.654 | 0.698 |
| 10^-3^ | 80115 | ZX | LM | -0.014 | 0.000 | 44.784 | 16.549 | 0.730 |
| 10^-4^ | 27385 | ZX | LM | -0.060 | 2.942 | 58.713 | 17.233 | 0.782 |
| 10^-5^ | 10898 | ZX | LM | -0.079 | 6.074 | 78.964 | 18.863 | 0.818 |
| 10^-6^ | 4148 | ZX | LM | -0.052 | 10.052 | 120.528 | 21.670 | 0.858 |
| 10^-7^ | 1945 | ZX | LM | -0.046 | 8.544 | 291.761 | 23.857 | 0.926 |
| 10^-8^ | 808 | ZX | LM | -0.056 | 14.411 | 546.162 | 23.875 | 0.959 |
| 10^-9^ | 449 | ZX | LM | 0.020 | 24.354 | 548.090 | 25.216 | 0.958 |
| 10^-1^ | 1356489 | LM | LM | 0.640 | 0.000 | 103.575 | 42.724 | 0.708 |
| 10^-2^ | 593467 | LM | LM | 0.636 | 0.000 | 90.614 | 43.748 | 0.674 |
| 10^-3^ | 280508 | LM | LM | 0.631 | 0.000 | 86.396 | 45.363 | 0.656 |
| 10^-4^ | 137439 | LM | LM | 0.626 | 2.735 | 82.573 | 47.148 | 0.644 |
| 10^-5^ | 66663 | LM | LM | 0.621 | 12.041 | 78.885 | 47.153 | 0.659 |
| 10^-6^ | 32921 | LM | LM | 0.619 | 20.780 | 83.643 | 47.712 | 0.686 |
| 10^-7^ | 16514 | LM | LM | 0.608 | 29.777 | 86.696 | 49.091 | 0.703 |
| 10^-8^ | 8763 | LM | LM | 0.595 | 37.008 | 84.311 | 50.390 | 0.707 |
| 10^-9^ | 4287 | LM | LM | 0.581 | 48.223 | 89.379 | 50.008 | 0.733 |

ref: reference population; tar: validation population;

cor: accuracy of genomic prediction;

varA: variance components accounted for by the remaining genome;

varE: variance components accounted for by the variants in the genomic feature;

varR: residual variance component.
