# Supplementary material for: Using imputation-based whole-genome sequencing data to improve the accuracy of genomic prediction for combined populations in pigs
Source: Genet Sel Evol. 2019 Oct 21;51:58. doi: 10.1186/s12711-019-0500-8 (PMC6805481; doi:10.1186/s12711-019-0500-8)
Supplement: Supplementary file 7 — Additional file 7: Tables S6. Accuracy of genomic prediction for backfat thickness (BFT) with different p values of GWAS as prior information in GFBLUP. [file 12711_2019_500_MOESM7_ESM.docx]

**Table S6.** The accuracy of genomic prediction for backfat thickness (BFT) with different p values of GWAS as prior information on GFBLUP.

| p-value | SNP number | ref | tar | cor | varA | varE | varR | $h^{2}$ |
| --- | --- | --- | --- | --- | --- | --- | --- | --- |
| 10^-1^ | 1212905 | LM+ZX | LM | 0.446 | 0.000 | 3.047 | 1.479 | 0.673 |
| 10^-2^ | 458875 | LM+ZX | LM | 0.437 | 0.000 | 2.723 | 1.495 | 0.646 |
| 10^-3^ | 186030 | LM+ZX | LM | 0.422 | 0.000 | 2.607 | 1.535 | 0.629 |
| 10^-4^ | 78470 | LM+ZX | LM | 0.405 | 0.034 | 2.593 | 1.571 | 0.626 |
| 10^-5^ | 32059 | LM+ZX | LM | 0.394 | 0.325 | 2.413 | 1.583 | 0.634 |
| 10^-6^ | 12642 | LM+ZX | LM | 0.395 | 0.428 | 2.606 | 1.613 | 0.653 |
| 10^-7^ | 4929 | LM+ZX | LM | 0.415 | 0.532 | 2.917 | 1.650 | 0.676 |
| 10^-8^ | 1820 | LM+ZX | LM | 0.452 | 0.663 | 3.691 | 1.696 | 0.720 |
| 10^-9^ | 618 | LM+ZX | LM | 0.433 | 1.134 | 2.945 | 1.735 | 0.702 |
| 10^-1^ | 1028035 | ZX | LM | 0.119 | 0.000 | 1.348 | 0.342 | 0.797 |
| 10^-2^ | 344848 | ZX | LM | 0.057 | 0.000 | 1.392 | 0.394 | 0.779 |
| 10^-3^ | 117607 | ZX | LM | 0.045 | 0.000 | 1.499 | 0.459 | 0.766 |
| 10^-4^ | 41558 | ZX | LM | 0.047 | 0.000 | 1.745 | 0.511 | 0.773 |
| 10^-5^ | 14980 | ZX | LM | 0.080 | 0.039 | 1.993 | 0.537 | 0.791 |
| 10^-6^ | 5162 | ZX | LM | 0.067 | 0.113 | 2.763 | 0.511 | 0.849 |
| 10^-7^ | 1965 | ZX | LM | 0.037 | 0.152 | 4.134 | 0.544 | 0.887 |
| 10^-8^ | 747 | ZX | LM | 0.055 | 0.343 | 4.381 | 0.575 | 0.891 |
| 10^-9^ | 98 | ZX | LM | -0.037 | 0.403 | 2.040 | 0.657 | 0.788 |
| 10^-1^ | 1239324 | LM | LM | 0.437 | 0.000 | 3.653 | 1.576 | 0.699 |
| 10^-2^ | 479616 | LM | LM | 0.433 | 0.000 | 3.318 | 1.592 | 0.676 |
| 10^-3^ | 197903 | LM | LM | 0.432 | 0.000 | 3.281 | 1.639 | 0.667 |
| 10^-4^ | 82603 | LM | LM | 0.416 | 0.140 | 3.129 | 1.683 | 0.660 |
| 10^-5^ | 33448 | LM | LM | 0.421 | 0.508 | 2.852 | 1.685 | 0.666 |
| 10^-6^ | 13835 | LM | LM | 0.432 | 0.593 | 3.356 | 1.698 | 0.699 |
| 10^-7^ | 5380 | LM | LM | 0.444 | 0.717 | 4.838 | 1.744 | 0.761 |
| 10^-8^ | 2403 | LM | LM | 0.439 | 0.737 | 8.745 | 1.815 | 0.839 |
| 10^-9^ | 1112 | LM | LM | 0.419 | 1.002 | 15.030 | 1.919 | 0.893 |

ref: reference population; tar: validation population;

cor: accuracy of genomic prediction;

varA: variance components accounted for by the remaining genome;

varE: variance components accounted for by the variants in the genomic feature;

varR: residual variance component.
