# Supplementary material for: Using imputation-based whole-genome sequencing data to improve the accuracy of genomic prediction for combined populations in pigs
Source: Genet Sel Evol. 2019 Oct 21;51:58. doi: 10.1186/s12711-019-0500-8 (PMC6805481; doi:10.1186/s12711-019-0500-8)
Supplement: Supplementary file 8 — Additional file 8: Tables S7. Accuracy of genomic prediction for number of piglets born alive (NBA) with different p values of GWAS as prior information in GFBLUP. [file 12711_2019_500_MOESM8_ESM.docx]

**Table S7.** The accuracy of genomic prediction for number of piglets born alive (NBA) with different p values of GWAS as prior information on GFBLUP.

| p-value | SNP number | ref | tar | cor | varA | varE | varR | $h^{2}$ |
| --- | --- | --- | --- | --- | --- | --- | --- | --- |
| 10^-1^ | 1408068 | LM+XD | LM | 0.462 | 0.000 | 0.980 | 0.414 | 0.703 |
| 10^-2^ | 554793 | LM+XD | LM | 0.454 | 0.000 | 0.885 | 0.417 | 0.680 |
| 10^-3^ | 237042 | LM+XD | LM | 0.441 | 0.000 | 0.898 | 0.434 | 0.674 |
| 10^-4^ | 108701 | LM+XD | LM | 0.411 | 0.054 | 1.045 | 0.456 | 0.707 |
| 10^-5^ | 52454 | LM+XD | LM | 0.393 | 0.165 | 1.224 | 0.457 | 0.752 |
| 10^-6^ | 25237 | LM+XD | LM | 0.374 | 0.235 | 1.534 | 0.463 | 0.793 |
| 10^-7^ | 12960 | LM+XD | LM | 0.376 | 0.312 | 1.862 | 0.472 | 0.822 |
| 10^-8^ | 6843 | LM+XD | LM | 0.392 | 0.369 | 2.276 | 0.482 | 0.846 |
| 10^-9^ | 3611 | LM+XD | LM | 0.392 | 0.470 | 1.905 | 0.498 | 0.827 |
| 10^-1^ | 1408068 | LM+XD | XD | 0.429 | 0.000 | 0.980 | 0.414 | 0.703 |
| 10^-2^ | 554793 | LM+XD | XD | 0.429 | 0.000 | 0.885 | 0.417 | 0.680 |
| 10^-3^ | 237042 | LM+XD | XD | 0.407 | 0.000 | 0.898 | 0.434 | 0.674 |
| 10^-4^ | 108701 | LM+XD | XD | 0.382 | 0.054 | 1.045 | 0.456 | 0.707 |
| 10^-5^ | 52454 | LM+XD | XD | 0.394 | 0.165 | 1.224 | 0.457 | 0.752 |
| 10^-6^ | 25237 | LM+XD | XD | 0.385 | 0.235 | 1.534 | 0.463 | 0.793 |
| 10^-7^ | 12960 | LM+XD | XD | 0.385 | 0.312 | 1.862 | 0.472 | 0.822 |
| 10^-8^ | 6843 | LM+XD | XD | 0.381 | 0.369 | 2.276 | 0.482 | 0.846 |
| 10^-9^ | 3611 | LM+XD | XD | 0.392 | 0.470 | 1.905 | 0.498 | 0.827 |
| 10^-1^ | 1153955 | XD | LM | -0.008 | 0.000 | 0.454 | 0.188 | 0.707 |
| 10^-2^ | 370659 | XD | LM | -0.010 | 0.000 | 0.377 | 0.194 | 0.660 |
| 10^-3^ | 122041 | XD | LM | -0.083 | 0.000 | 0.328 | 0.208 | 0.612 |
| 10^-4^ | 41555 | XD | LM | -0.088 | 0.000 | 0.317 | 0.224 | 0.585 |
| 10^-5^ | 13428 | XD | LM | -0.078 | 0.000 | 0.322 | 0.238 | 0.574 |
| 10^-6^ | 4105 | XD | LM | -0.045 | 0.000 | 0.370 | 0.249 | 0.598 |
| 10^-7^ | 1386 | XD | LM | 0.057 | 0.032 | 0.451 | 0.252 | 0.657 |
| 10^-8^ | 479 | XD | LM | 0.089 | 0.033 | 0.542 | 0.291 | 0.665 |
| 10^-9^ | 164 | XD | LM | -0.121 | 0.062 | 0.575 | 0.315 | 0.670 |
| 10^-1^ | 1153955 | XD | XD | 0.378 | 0.000 | 0.454 | 0.188 | 0.707 |
| 10^-2^ | 370659 | XD | XD | 0.373 | 0.000 | 0.377 | 0.194 | 0.660 |
| 10^-3^ | 122041 | XD | XD | 0.368 | 0.000 | 0.328 | 0.208 | 0.612 |
| 10^-4^ | 41555 | XD | XD | 0.369 | 0.000 | 0.317 | 0.224 | 0.585 |
| 10^-5^ | 13428 | XD | XD | 0.356 | 0.000 | 0.322 | 0.238 | 0.574 |
| 10^-6^ | 4105 | XD | XD | 0.330 | 0.000 | 0.389 | 0.249 | 0.610 |
| 10^-7^ | 1386 | XD | XD | 0.346 | 0.032 | 0.451 | 0.252 | 0.657 |
| 10^-8^ | 479 | XD | XD | 0.337 | 0.033 | 0.552 | 0.291 | 0.668 |
| 10^-9^ | 164 | XD | XD | 0.262 | 0.062 | 0.633 | 0.315 | 0.689 |
| 10^-1^ | 1396538 | LM | LM | 0.464 | 0.000 | 1.289 | 0.451 | 0.741 |
| 10^-2^ | 544905 | LM | LM | 0.455 | 0.000 | 1.166 | 0.459 | 0.718 |
| 10^-3^ | 233293 | LM | LM | 0.428 | 0.000 | 1.267 | 0.478 | 0.726 |
| 10^-4^ | 109387 | LM | LM | 0.421 | 0.158 | 1.443 | 0.494 | 0.764 |
| 10^-5^ | 53341 | LM | LM | 0.405 | 0.273 | 1.805 | 0.496 | 0.807 |
| 10^-6^ | 26603 | LM | LM | 0.408 | 0.379 | 2.234 | 0.498 | 0.840 |
| 10^-7^ | 13917 | LM | LM | 0.413 | 0.475 | 2.695 | 0.513 | 0.861 |
| 10^-8^ | 7236 | LM | LM | 0.390 | 0.558 | 3.132 | 0.539 | 0.873 |
| 10^-9^ | 4212 | LM | LM | 0.434 | 0.624 | 4.327 | 0.552 | 0.900 |
| 10^-1^ | 1396538 | LM | XD | 0.221 | 0.000 | 1.289 | 0.451 | 0.741 |
| 10^-2^ | 544905 | LM | XD | 0.206 | 0.000 | 1.166 | 0.459 | 0.718 |
| 10^-3^ | 233293 | LM | XD | 0.168 | 0.000 | 1.267 | 0.478 | 0.726 |
| 10^-4^ | 109387 | LM | XD | 0.144 | 0.158 | 1.443 | 0.494 | 0.764 |
| 10^-5^ | 53341 | LM | XD | 0.151 | 0.273 | 1.805 | 0.496 | 0.807 |
| 10^-6^ | 26603 | LM | XD | 0.207 | 0.379 | 2.234 | 0.498 | 0.840 |
| 10^-7^ | 13917 | LM | XD | 0.173 | 0.475 | 2.695 | 0.513 | 0.861 |
| 10^-8^ | 7236 | LM | XD | 0.164 | 0.558 | 3.132 | 0.539 | 0.873 |
| 10^-9^ | 4212 | LM | XD | 0.146 | 0.624 | 4.327 | 0.552 | 0.900 |

ref: reference population; tar: validation population;

cor: accuracy of genomic prediction;

varA: variance components accounted for by the remaining genome;

varE: variance components accounted for by the variants in the genomic feature;

varR: residual variance component.
