# Supplementary material for: Using imputation-based whole-genome sequencing data to improve the accuracy of genomic prediction for combined populations in pigs
Source: Genet Sel Evol. 2019 Oct 21;51:58. doi: 10.1186/s12711-019-0500-8 (PMC6805481; doi:10.1186/s12711-019-0500-8)
Supplement: Supplementary file 9 — Additional file 9: Tables S8. Accuracy of genomic prediction for total number of piglets born (TNB) with different p values of GWAS as prior information in GFBLUP. [file 12711_2019_500_MOESM9_ESM.docx]

**Table S8.** The accuracy of genomic prediction for total number of piglets born (TNB) with different p values of GWAS as prior information on GFBLUP.

| p-value | SNP number | ref | tar | cor | varA | varE | varR | $h^{2}$ |
| --- | --- | --- | --- | --- | --- | --- | --- | --- |
| 10^-1^ | 1419825 | LM+XD | LM | 0.455 | 0.000 | 1.348 | 0.529 | 0.718 |
| 10^-2^ | 560256 | LM+XD | LM | 0.448 | 0.000 | 1.202 | 0.541 | 0.690 |
| 10^-3^ | 237134 | LM+XD | LM | 0.435 | 0.000 | 1.161 | 0.567 | 0.672 |
| 10^-4^ | 106843 | LM+XD | LM | 0.423 | 0.123 | 1.282 | 0.583 | 0.707 |
| 10^-5^ | 50828 | LM+XD | LM | 0.409 | 0.269 | 1.521 | 0.583 | 0.754 |
| 10^-6^ | 24754 | LM+XD | LM | 0.405 | 0.402 | 1.817 | 0.584 | 0.792 |
| 10^-7^ | 12341 | LM+XD | LM | 0.404 | 0.500 | 2.191 | 0.594 | 0.819 |
| 10^-8^ | 6299 | LM+XD | LM | 0.377 | 0.608 | 2.630 | 0.594 | 0.845 |
| 10^-9^ | 3025 | LM+XD | LM | 0.359 | 0.700 | 2.505 | 0.620 | 0.838 |
| 10^-1^ | 1419825 | LM+XD | XD | 0.485 | 0.000 | 1.348 | 0.529 | 0.718 |
| 10^-2^ | 560256 | LM+XD | XD | 0.494 | 0.000 | 1.202 | 0.541 | 0.690 |
| 10^-3^ | 237134 | LM+XD | XD | 0.481 | 0.000 | 1.161 | 0.567 | 0.672 |
| 10^-4^ | 106843 | LM+XD | XD | 0.466 | 0.123 | 1.282 | 0.583 | 0.707 |
| 10^-5^ | 50828 | LM+XD | XD | 0.469 | 0.269 | 1.521 | 0.583 | 0.754 |
| 10^-6^ | 24754 | LM+XD | XD | 0.454 | 0.402 | 1.817 | 0.584 | 0.792 |
| 10^-7^ | 12341 | LM+XD | XD | 0.417 | 0.500 | 2.191 | 0.594 | 0.819 |
| 10^-8^ | 6299 | LM+XD | XD | 0.409 | 0.608 | 2.630 | 0.594 | 0.845 |
| 10^-9^ | 3025 | LM+XD | XD | 0.431 | 0.700 | 2.505 | 0.620 | 0.838 |
| 10^-1^ | 1219182 | XD | LM | -0.064 | 0.000 | 0.639 | 0.179 | 0.781 |
| 10^-2^ | 402480 | XD | LM | -0.055 | 0.000 | 0.508 | 0.184 | 0.734 |
| 10^-3^ | 140643 | XD | LM | -0.098 | 0.000 | 0.448 | 0.202 | 0.689 |
| 10^-4^ | 48583 | XD | LM | -0.109 | 0.000 | 0.388 | 0.230 | 0.628 |
| 10^-5^ | 17265 | XD | LM | -0.050 | 0.000 | 0.413 | 0.242 | 0.630 |
| 10^-6^ | 5911 | XD | LM | -0.048 | 0.041 | 0.425 | 0.243 | 0.657 |
| 10^-7^ | 1904 | XD | LM | -0.049 | 0.080 | 0.355 | 0.253 | 0.632 |
| 10^-8^ | 649 | XD | LM | 0.009 | 0.066 | 0.384 | 0.288 | 0.609 |
| 10^-9^ | 221 | XD | LM | -0.123 | 0.076 | 0.543 | 0.321 | 0.659 |
| 10^-1^ | 1219182 | XD | XD | 0.422 | 0.000 | 0.639 | 0.179 | 0.781 |
| 10^-2^ | 402480 | XD | XD | 0.420 | 0.000 | 0.508 | 0.184 | 0.734 |
| 10^-3^ | 140643 | XD | XD | 0.414 | 0.000 | 0.448 | 0.202 | 0.689 |
| 10^-4^ | 48583 | XD | XD | 0.432 | 0.000 | 0.388 | 0.230 | 0.628 |
| 10^-5^ | 17265 | XD | XD | 0.425 | 0.000 | 0.413 | 0.242 | 0.630 |
| 10^-6^ | 5911 | XD | XD | 0.398 | 0.041 | 0.425 | 0.243 | 0.657 |
| 10^-7^ | 1904 | XD | XD | 0.404 | 0.080 | 0.355 | 0.253 | 0.632 |
| 10^-8^ | 649 | XD | XD | 0.389 | 0.066 | 0.384 | 0.288 | 0.609 |
| 10^-9^ | 221 | XD | XD | 0.369 | 0.076 | 0.543 | 0.321 | 0.659 |
| 10^-1^ | 1410194 | LM | LM | 0.458 | 0.000 | 1.789 | 0.598 | 0.750 |
| 10^-2^ | 544820 | LM | LM | 0.459 | 0.000 | 1.632 | 0.612 | 0.727 |
| 10^-3^ | 226150 | LM | LM | 0.439 | 0.038 | 1.603 | 0.649 | 0.717 |
| 10^-4^ | 99201 | LM | LM | 0.417 | 0.301 | 1.765 | 0.655 | 0.759 |
| 10^-5^ | 47437 | LM | LM | 0.401 | 0.437 | 2.260 | 0.654 | 0.805 |
| 10^-6^ | 22383 | LM | LM | 0.400 | 0.558 | 2.880 | 0.658 | 0.839 |
| 10^-7^ | 11496 | LM | LM | 0.403 | 0.689 | 3.608 | 0.664 | 0.866 |
| 10^-8^ | 4506 | LM | LM | 0.394 | 0.830 | 3.138 | 0.689 | 0.852 |
| 10^-9^ | 1864 | LM | LM | 0.405 | 0.878 | 2.876 | 0.711 | 0.841 |
| 10^-1^ | 1410194 | LM | XD | 0.267 | 0.000 | 1.789 | 0.598 | 0.750 |
| 10^-2^ | 544820 | LM | XD | 0.273 | 0.000 | 1.632 | 0.612 | 0.727 |
| 10^-3^ | 226150 | LM | XD | 0.282 | 0.038 | 1.603 | 0.649 | 0.717 |
| 10^-4^ | 99201 | LM | XD | 0.228 | 0.301 | 1.765 | 0.655 | 0.759 |
| 10^-5^ | 47437 | LM | XD | 0.199 | 0.437 | 2.260 | 0.654 | 0.805 |
| 10^-6^ | 22383 | LM | XD | 0.130 | 0.558 | 2.880 | 0.658 | 0.839 |
| 10^-7^ | 11496 | LM | XD | 0.048 | 0.689 | 3.608 | 0.664 | 0.866 |
| 10^-8^ | 4506 | LM | XD | 0.013 | 0.830 | 3.138 | 0.689 | 0.852 |
| 10^-9^ | 1864 | LM | XD | 0.028 | 0.878 | 2.876 | 0.711 | 0.841 |

ref: reference population; tar: validation population;

cor: accuracy of genomic prediction;

varA: variance components accounted for by the remaining genome;

varE: variance components accounted for by the variants in the genomic feature;

varR: residual variance component.
